# Advances in RNAi-Assisted Strain Engineering in *Saccharomyces cerevisiae*

**DOI:** 10.3389/fbioe.2020.00731

**Published:** 2020-07-02

**Authors:** Yongcan Chen, Erpeng Guo, Jianzhi Zhang, Tong Si

**Affiliations:** CAS Key Laboratory of Quantitative Engineering Biology, Shenzhen Institute of Synthetic Biology, Shenzhen Institutes of Advanced Technology, Chinese Academy of Sciences, Shenzhen, China

**Keywords:** RNAi, *Saccharomyces cerevisiae*, strain engineering, functional genomics, synthetic biology

## Abstract

*Saccharomyces cerevisiae* is a widely used eukaryotic model and microbial cell factory. RNA interference (RNAi) is a conserved regulatory mechanism among eukaryotes but absent from *S. cerevisiae*. Recent reconstitution of RNAi machinery in *S. cerevisiae* enables the use of this powerful tool for strain engineering. Here we first discuss the introduction of heterologous RNAi pathways in *S. cerevisiae*, and the design of various expression cassettes of RNAi precursor reagents for tunable, dynamic, and genome-wide regulation. We then summarize notable examples of RNAi-assisted functional genomics and metabolic engineering studies in *S. cerevisiae*. We conclude with the future challenges and opportunities of RNAi-based approaches, as well as the potential of other regulatory RNAs in advancing yeast engineering.

## Introduction

As a unicellular eukaryotic microorganism, *S. cerevisiae* has become one of the most widely used microbial cell factory in the production of value-added chemicals, biofuels and biopharmaceuticals (Liu et al., 2013; Nielsen, 2019). *S. cerevisiae* has many desirable traits for industrial fermentation. With a long history in baking and brewing, *S. cerevisiae* is generally recognized as safe (GRAS). Compared with prokaryotes such as *E. coli*, *S. cerevisiae* possesses multiple organelles providing physical compartments for diverse biochemical reactions; it is also capable of post-translational modifications, which are required for heterologous synthesis of complex proteins (Tokmakov et al., 2012). *S. cerevisiae* is physiologically stable and highly robust toward harsh industrial conditions such as low pH, high osmotic pressure, and toxic inhibitors (Hong and Nielsen, 2012; Kavscek et al., 2015). Due to its popularity in fundamental and applied research, versatile tools have been developed in *S. cerevisiae* for genetic manipulation (Si et al., 2015b; Lian et al., 2018; Jiang et al., 2019) and bioprocess development (Hasunuma and Kondo, 2012; Tripathi and Shrivastava, 2019).

RNA interference (RNAi) is a post-transcriptional, gene-silencing mechanism broadly distributed in eukaryotic organisms. RNAi mediates many essential biological processes, including defense against viruses and transposons, maintenance of chromosome and genome integrity, and cellular differentiation and development (Castel and Martienssen, 2013; Gutbrod and Martienssen, 2020). Various organisms comprise different mechanisms for RNAi, but the basic process shared three common steps. First, small interfering RNA (siRNA) duplexes of 21–25 nucleotides are generated from long double-stranded RNA (dsRNA) precursors by a ribonuclease enzyme called Dicer. Second, siRNAs are loaded into Argonaute proteins to form a protein-RNA complex known as RNA-induced silencing complex (RISC), where the guide and passenger strands of siRNA are dissociated. Third, RISC finds and cleaves the cognate mRNA molecule, whose sequence is homologous to the siRNA loaded in the complex (Hannon, 2002; Wilson and Doudna, 2013; Ipsaro and Joshua-Tor, 2015). Thanks to its facile implementation and high specificity, RNAi has been widely used to knock down individual genes of interest, as well as to perform genome-wide reduction-of-function screening, since its first discovery in *Caenorhabditis elegans* (Fire et al., 1998; Agrawal et al., 2003; Boutros and Ahringer, 2008).

RNA interference is conserved in almost all eukaryotic organisms including fungi, plants and animals (Agrawal et al., 2003; Gutbrod and Martienssen, 2020), but it is evolutionarily lost in *S. cerevisiae*, possibly due to the incompatibility with a beneficial dsRNA “killer virus” (Drinnenberg et al., 2009, 2011). This theory is supported by the ecological significance of “killer viruses” (Boynton, 2019) and the importance of RNAi in antiviral defense (Li et al., 2013; Waldron et al., 2018). However, a non-canonical RNAi pathway exists in other budding yeasts, including *Saccharomyces castellii*, *Candida albicans*, and *Kluyveromyces polysporus* (Drinnenberg et al., 2009). Unlike canonical Dicers, which generate siRNAs of regular sizes from dsRNA termini, budding-yeast Dicers start processing in the interior of a dsRNA and work outward, with product size determined by the distance between neighboring active sites (Weinberg et al., 2011; Wilson and Doudna, 2013). On the other hand, other yeasts contain canonical RNAi machinery, including the fission yeast *Schizosaccharomyces pombe* (Volpe et al., 2002) and a human pathogenic yeast *Cryptococcus neoformans* (Loftus et al., 2005).

Since the discovery and characterization of the budding yeast pathways, RNAi has become an attractive platform for gene regulation in *S. cerevisiae*. Here we summarize the reconstitution of heterologous RNAi pathways in baker’s yeast and the design of RNAi cassettes for desirable potency and specificity. We also provide notable examples in implementing RNA-based engineering tools for tunable, dynamic, and genome-scale gene modulation in *S. cerevisiae*. To conclude, the future directions of RNAi-based tools and the potentials of other regulatory RNAs in *S. cerevisiae* strain engineering are discussed.

## Tool Development

### RNAi Pathway Reconstitution

Heterologous RNAi pathways from *S. castelli* and human were successfully reconstituted in *S. cerevisiae*. To evaluate RNAi effectiveness and efficiency, fluorescent proteins were commonly utilized as reporters (Drinnenberg et al., 2009; Suk et al., 2011; Crook et al., 2014; Si et al., 2014; Purcell et al., 2018; Kildegaard et al., 2019). For the *S. castelli* pathway, robust repression of green fluorescent protein (GFP) signals were observed when both *DCR1* and *AGO1* were present, but *DCR1* alone was sufficient to generate GFP siRNAs in *S. cerevisiae* (Drinnenberg et al., 2009). On the other hand, all three components of the human RISC complex, Dicer, Argonaute-2 (Ago2), and HIV-1 transactivating response (TAR) RNA-binding protein (TRBP) (Gregory et al., 2005; Maniataki and Mourelatos, 2005), are necessary to reconstitute functional RNAi in *S. cerevisiae* (Suk et al., 2011). This difference between *S. castelli* and human pathways is probably because human TRBP is required to mediate interaction of Ago2 and siRNA bounded by Dicer, whereas budding yeast Dicers act as homodimers and did not require additional dsRBD domains (Chendrimada et al., 2005; Weinberg et al., 2011).

**FIGURE 1:**
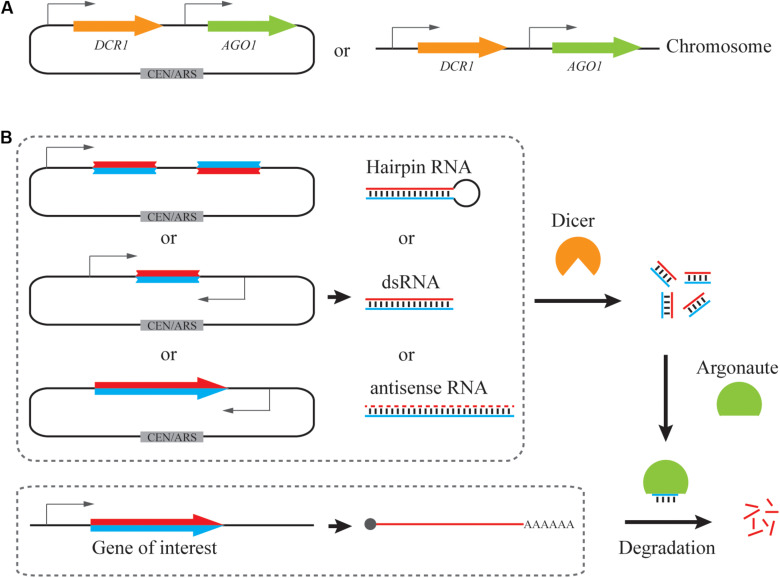
Reconstitution of a synthetic RNAi pathway in *S. cerevisiae*. **(A)** Expression cassettes of RNAi pathway. *DCR1* and *AGO1* from *S. castellii* are more commonly utilized, being cloned into a low-copy plasmid or integrated into the genome. **(B)** Expression cassettes of siRNA precursors. Hairpin RNA, dsRNA and full-length antisense RNA have been used for generating siRNAs by Dicer. siRNA duplexes mediate mRNA degradation and hence repression of a target gene with the help of Argonaute.

Due to evolutionary closeness and simplicity, the *S. castellii* RNAi pathway is commonly employed in *S. cerevisiae* (Drinnenberg et al., 2009; Crook et al., 2014; Si et al., 2014; Williams et al., 2015b; Purcell et al., 2018; Wang et al., 2019; Figure 1A). *S. castellii* Dicer and Argonaute were expressed either from a low-copy (centromeric) plasmid (Crook et al., 2014, 2016; Lee et al., 2016) or a single, integrated locus in the yeast genome (Drinnenberg et al., 2009; Si et al., 2014, 2017; Xiao and Zhao, 2014; Williams et al., 2015a, b; Purcell et al., 2018; Wang et al., 2019; Figure 1A and Table 1). To enable efficient gene silencing, gene expression of Dicer and Argonaute was driven by strong constitutive promoters, such as P_TEF1_, P_GPD1_, P_TPI1_, and P_PGK1_. Although no comprehensive comparisons have been performed among different expression formats, it is suggested that constitutive expression driven by a strong promoter from a genomic locus is sufficient for efficient RNAi silencing.

**TABLE 1:** Expression cassettes and efficiency of RNAi in *S. cerevisiae.*

siRNA precursor	Cassettes for RNAi		Silencing efficiency	References
	
	Pathway	siRNA precursor		
hairpin RNA	pRS404-TEF1p-Ago1 and pRS405-TEF1p-Dcr1	pRS403-GAL1p-GFP (275 bp)-*RAD9* intron 1-GFPrc	strong silencing	Drinnenberg et al., 2009
	p415-TDH3p-Dicer-TEF1p-Ago2	pRS414-TDH3p-YFP (200 bp)-intron-YFPrc	93% and 80% for highly and weakly expressed genome-integrated YFP	Crook et al., 2014
	pRS404-PTEF-Ago1(x2)-pRS405-PTEF-Dcr1(x2)	PFUS1J2- ARO7sense-rad9linker- ARO7antisense-pRS413; PFUS1J2-ZWF1/CDC19sense-rad9linker-ZWF1/CDC19antisense- pRS406	97% for *ARO7*, 87.8% for *CDC19*, insignificant for *ZWF1*	Williams et al., 2015a
	pX-3-AGO1-DCR1 TDH3p:ZWF1↓:can1; pX-3-AGO1-DCR1 RNR2p:ZWF1↓:can1	pX-3-AGO1-DCR1 TDH3p:ZWF1↓:can1; pX-3-AGO1-DCR1 RNR2p:ZWF1↓:can1	95% or 80% for TDH3p or RNR2p-drived shRNA expression, respectively	Kildegaard et al., 2019
dsRNA	pRS404-TEF1p-Ago1 and pRS405-TEF1p-Dcr1	pRS403-GAL1p-GFP (275 bp)-URA3prc	intermediate silencing	Drinnenberg et al., 2009
	pRS-delta-KanMX-LoxP-TEF1p-AGO1-PGK1t-TPI1p-DCR1-GPD1t	pRS416-GPDtrc-TEF1p-GFP (1–180 bp region)-TPI1prc- PGK1t;	∼80%	Si et al., 2014
	p415-TDH3p-Dicer-TEF1p-Ago2	pRS414-TDH3p-*RAD9* intron-YFP (200 bp)-*RAD9* intron rc-TEF1prc	84% for strongly expressed YFP, 94% for weakly expressed YFP	Crook et al., 2016
antisense RNA	pAG413Gal-Ago2, pAG416Gal-Dicer, pAG415Gal-TRBP	pAG424Gal-AS-GFP	80%	Suk et al., 2011
	pRS-delta-KanMX-LoxP-TEF1p-AGO1 -PGK1t-TPI1p-DCR1-GPD1t	pRS406-TEF1p-AS GFP-PGK1t	∼95%	Si et al., 2014

### RNAi Reagent Cassette Design

In addition to RNAi pathways, RNAi precursor is another essential component for efficient gene silencing. We will limit our discussions to the design of RNAi reagent cassettes for the *S. castellii* RNAi pathway, which is widely employed for RNAi reconstitution in *S. cerevisiae*. In its native host, *S. castellii* siRNAs are originated from hairpin RNAs, which are transcribed from inverted genomic repeats and fold back on their own paired regions (Drinnenberg et al., 2009). These RNA hairpins were presumed to be composed of stems of 100–400 bp and loops ranging from 19 to >1600 nt (Drinnenberg et al., 2009). As expected, when co-expressed with *S. castellii* Dcr1 and Ago1 in *S. cerevisiae*, the hairpin RNA construct with inverted repeats of a 275 bp GFP fragment induced strong reduction of GFP mRNA and fluorescence levels (Drinnenberg et al., 2009). In addition to hairpin RNAs (Drinnenberg et al., 2009; Crook et al., 2014; Purcell et al., 2018), other forms of RNAi precursors, such as dsRNAs (Si et al., 2014; Crook et al., 2016) and antisense RNAs (Suk et al., 2011; Si et al., 2014, 2017), can also be accepted by the *S. castellii* RNA machinery for gene repression in *S. cerevisiae*. Notably, although a growing collection of computational tools is available to design RNAi targeting sequences, these tools are primarily developed for canonical RNAi pathways as recently summarized (Lagana et al., 2014; Jain and Wadhwa, 2018). Given the mechanistic differences of siRNA generation between canonical and budding-yeast RNAi machinery, it remains elusive if existing algorithms may help to identify RNAi targets for efficient gene silencing in *S. cerevisiae*. Here, we will focus on how different designs of expression cassettes may result in various levels of RNAi silencing (Table 1).

#### Hairpin RNA

The design of hairpin RNA cassettes, such as promoter, length of hairpin RNAs, and plasmid copy number, has a major impact on RNAi silencing efficiency (Table 1). To state a general observation, a low-copy auxotrophic plasmid expressing long hairpin by a strong promoter produces a potent RNAi reagent.

To investigate the impact of promoter strength, Crook et al. engineered reporter strains expressing yellow fluorescence protein (YFP) from a strong or weak promoter, which could mimic the different expression levels of native genes. Only when the hairpin RNA was expressed from the strong P_TDH3_ promoter, efficient repression (up to 80%) was obtained for strongly expressed YFP (Crook et al., 2014), indicating the amount of RNAi precursor was limited in this case. Moreover, three promoters with different strength (P_CYC1_, P_TEF1_ and P_TDH3_) were used to express a hairpin RNA targeting *ADE3*, the deletion of which can improve heterologous production of itaconic acid (IA). Various IA production levels were observed with different levels of hairpin RNA expression in three *S. cerevisiae* strains (BY4741, CEN.PK2-a, and Sigma 10560-4A), demonstrating the necessity of promoter screening to fine-tune RNAi efficacy (Crook et al., 2014). Similarly, the strong P_TDH3_ promoter elicited higher repression levels relative to the weak P_RNR2_ promoter when targeting the endogenous *ZWF1* gene using hairpin RNAi (Kildegaard et al., 2019). The observation that the promoter activity of a hairpin RNA cassette modulates its gene silencing efficiency was exploited for dynamic, context-dependent gene knockdown, whereby an inducible promoter was used to drive hairpin RNA expression (Williams et al., 2015a).

Hairpin length also affects RNAi efficiency. When the length of hairpin increased from 100 bp to 200 bp, the downregulation efficiency was improved by 30% when YFP is strongly expressed (Crook et al., 2014). Notably, when YFP was weakly expressed, the inhibition level of 200 bp hairpin was 6-fold higher than that of the 100 bp hairpin, indicating that longer hairpin length is especially important for efficient silencing of low-abundance transcripts. The potency of long (∼250 bp) RNAi hairpins in *S. cerevisiae* was also confirmed in later studies (Williams et al., 2015a; Kildegaard et al., 2019).

For plasmid copy number, when using an auxotrophic marker (*TRP1*), a low-copy, centromeric plasmid achieved higher knockdown efficiency relative to a high-copy, 2-micron vector, for both strongly and weakly expressed YFP (Crook et al., 2014). This effect, however, cannot be observed when using an antibiotic resistance marker (KanMX). Improved RNAi efficiency by low-copy, centromeric plasmid may be related to reduced cell-to-cell variability.

It is also possible to introduce synthetic RNAi targets to a native transcript. A single, invariant target sequence was inserted into the 3′ untranslated region (UTR) of a yERFP reporter gene, and repression of fluorescence signal was achieved by introducing a corresponding hairpin RNA in an RNAi capable *S. cerevisiae* (Purcell et al., 2018). Gene silencing efficiency can be modulated via variations in the repeat number or sequence length of targeting siRNA sequences placed in the stem structure. For example, more repeats induced stronger gene repression. The authors also demonstrated that nucleus-residing non-coding RNA can be targeted by hairpin RNAs, but it was unlikely mediated by RNAi, because RNAi effector proteins are normally expressed in cytosol. In this way, any gene can be theoretically regulated by the same RNAi precursor upon insertion of a synthetic target sequence in the UTRs, offering a graded and scalable module of gene regulation.

#### Long dsRNA

Long dsRNA molecules without hairpin loop structures can also be used as siRNA precursors. The dsRNA molecules can be transcribed by convergent promoters, whereby the sense and antisense transcripts are individually expressed under the control of two opposing promoters. However, it was showed that the amount of siRNA from dsRNA was much lower than from hairpin RNA precursors, leading to weaker GFP repression (Drinnenberg et al., 2009). Like hairpin RNA, strong promoters achieved higher repression than weaker promoters when driving dsRNA expression (Crook et al., 2016). To investigate the influence of dsRNA length, we generated various dsRNA constructs on a low-copy, centromeric plasmid (pRS416) targeting different regions of the *GFP* gene, driven by two strong, convergent promoters P_TEF1_ and P_TPI1_. Among the dsRNAs corresponding to the 1–180, 1–360, 1–540, and 1–717 bp (full length) region of the *GFP* gene, knockdown efficiency was inversely related to dsRNA length, with the shortest (1–180 bp) dsRNA exhibited the strongest GFP repression (80%) (Si et al., 2014). As to the vector of dsRNA cassette, although efficient downregulation was observed from a centromeric plasmid, the silencing efficiency was even higher when integrated into the genome (Si et al., 2014; Crook et al., 2016). In addition, with the introduction of an intron from *Schizosaccharomyces pombe RAD9* directly downstream of convergent promoters to enhance transcriptional strength, the extent of downregulation was significantly improved for weakly expressed YFP, but insignificant for strongly expressed YFP (Crook et al., 2016). Together, similar to hairpin RNA, the use of strong promoters and low-copy vectors is desirable for dsRNA. The length of dsRNAs should be carefully optimized, and ∼200 bp was frequently used (Si et al., 2014; Crook et al., 2016; Table 1).

#### Antisense RNA

Antisense RNAs can act as siRNA precursors by hybridizing with endogenous transcripts to form dsRNA substrates of Dicer. We expressed the full-length, antisense GFP transcript under control of the P_TEF1_ promoter on an episomal plasmid. Silencing effect is negligible without a RNA pathway, and a 95% repression of GFP signal was observed in an RNAi capable strain (Si et al., 2014; Table 1). When integrated into the genome, an even higher silencing efficiency and less population heterogeneity in fluorescence profile were observed (Si et al., 2014).

Taken together, hairpin RNA, dsRNA, and antisense RNA have been employed for RNAi silencing in *S. cerevisiae* (Figure 1B). Upon optimization, all three forms of siRNA precursor could induce strong gene knockdown efficiency (up to 95%). However, the design of hairpin RNA and dsRNA expression cassettes need more optimization, including the length and region of the targeting sequence. In addition, hairpin RNA structures with longer inverted repeats are difficult to build and unstable during cloning, probably due to interference with DNA replication (Voineagu et al., 2008). An intron-containing gap region could help to improve plasmid stability (Drinnenberg et al., 2009; Crook et al., 2014). Alternatively, full-length antisense RNA represents a simple yet effective RNAi cassette design.

### Genome-Scale Library Design

**FIGURE 2:**
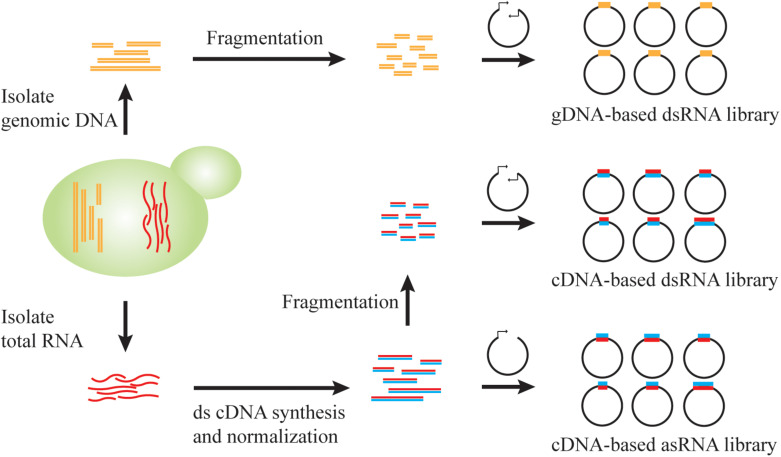
Genome-scale RNAi library construction in *S. cerevisiae*. RNAi library can be constructed from genomic DNA (gDNA) or total RNA. By cloning the fragmented genomic DNA into a vector with convergent promoters, a gDNA-based dsRNA library enables the generation of dsRNA precursors for RNAi pathway. After normalization of transcript abundance, the double-stranded cDNA could be fragmented and cloned into a vector with convergent promoters for a dsRNA library or be directionally cloned into a vector for antisense RNA transcription.

A remarkable advantage of RNAi technology is genome-wide reduction-of-function screening (Boutros and Ahringer, 2008; Mohr et al., 2014). Genome-scale coverage, knockdown efficiency, and off-target effects are important considerations when designing large-scale RNAi experiments (Boutros and Ahringer, 2008; Horn et al., 2010). For heterologous RNAi in *S. cerevisiae*, these characteristics are related to the form of siRNA precursors, the DNA source of RNAi cassettes, the length of inserted fragments, and the library size (Figure 2). Due to the difficulty in construction of long inverted repeats and the issue of plasmid stability, it is challenging to generate genome-wide RNAi libraries based on hairpin RNAs. Alternatively, dsRNA and antisense RNA are commonly utilized to create comprehensive RNAi libraries in *S. cerevisiae*.

Pooled long-dsRNA libraries can be created by inserting genomic DNA or complementary DNA (cDNA) fragments between convergent promoters (Cheng and Jian, 2010; Si et al., 2014). Genomic DNA or cDNA fragments can be generated either through endonuclease-mediated, biochemical digestion, such as *Sau*3AI (Si et al., 2014), or using physical shearing method, such as ultrasonication (Crook et al., 2016). Fragmentation parameters should be carefully tuned to achieve a proper length distribution. For example, we previously constructed a strain library of >3 × 10^5^ independent clones, where each clone contained ∼200 bp genomic DNA fragments. This library achieved a >5-fold redundancy of the 12 Mb yeast genome and hence >99% probability of a full genomic coverage (Si et al., 2014). Also, double-stranded cDNA were fragmented by ultrasonication to an average of 200 or 400 bp to minimize possible translation of open reading frames within dsRNA constructs (Crook et al., 2016). The resultant library contained over 10^5^ distinct members and achieved a good coverage of yeast transcriptome (Lee et al., 2016). Notably, due to the heterogeneity of transcript abundance, cDNA normalization via selective degradation of abundant transcripts is necessary to improve genome-scale representation (Zhulidov et al., 2004; Si et al., 2017). On the other hand, genomic DNA-derived dsRNA libraries exhibit an even coverage of each gene, but such libraries will also contain non-transcribed regions that may be inefficient RNAi reagents in most cases. However, this feature may reveal unexpected RNAi targets such as tRNAs (Si et al., 2014), which are not commonly included in cDNA libraries.

Alternatively, antisense RNA libraries can be generated by cloning of cDNA molecules in the reverse direction downstream of a promoter. Based on the SMART (Switching Mechanism At 5′-end of RNA Template) mechanism (Zhu et al., 2001), two different adaptor sequences are included specifically to each end of a full-length cDNA molecule, and hence insertion direction can be controlled by arranging the homologous adaptor sequences in a desirable order in an expression cassette (Si et al., 2017). Upon cDNA normalization based on transcript abundance, a strain library of >10^6^ independent clones achieved >92% coverage of all yeast genes, which was confirmed via next generation sequencing (NGS) analysis (Si et al., 2017). Taken together, genomic DNA and cDNA libraries can be readily sourced for generating RNAi reagents for genome-wide, reduction-of-function screening in *S. cerevisiae*.

## Application

As one of the most widely used cell factory, *S. cerevisiae* is intensively engineered with different industrially relevant traits, including broadened substrate scope, improved titer, yield and productivity, and enhanced robustness (Kavscek et al., 2015; Lian et al., 2018). Due to the complexity of cellular networks, strain engineering requires concerted identification of genetic targets on a genome scale and fine-tuning of their expression with temporal precision (Si et al., 2015a; Lian et al., 2018). As a modular, scalable, and predictable tool for gene regulation, RNAi has been increasingly applied in yeast engineering, offering unique advantages such as dynamic regulation, genome-scale engineering, and multigene optimization.

### Dynamic Regulation

Introduction of synthetic constructs and heterologous pathways typically causes metabolic burden, due to competition for cellular resources and accumulation of toxic intermediates and products (Keasling, 2008; Borkowski et al., 2016). To address this problem, the growth and production phases can be separated via dynamic gene regulation, which can be achieved by inducible RNAi expression.

GAL and Tet are well-characterized inducible promoters in *S. cerevisiae*, modulated by galactose and doxycycline, respectively (Blount et al., 2012). As a proof of concept, when a hairpin RNA cassette targeting *URA3* was constructed under the control of P_GAL1_, addition of galactose reduced cell growth in the absence of uracil, and enabled cell growth in the presence of 5-fluoroorotic acid (5-FOA), which can be converted to a toxic product by Ura3p (Drinnenberg et al., 2009). Inducible expression of RNAi hairpins were also demonstrated using P_TPGI_, a promoter derived from P_GAL1_ and induced by galactose and anhydrotetracycline (aTc) (Purcell et al., 2018). Although dynamic RNAi regulation was achieved, the use of GAL promoters requires expensive inducers and suffers from leaky expression. Therefore, it is desirable to utilize promoters that are strongly repressed by glucose and activated by a cheap carbon source. Williams et al. found that during long-term fermentation, GFP expression from the *SUC2* promoter was strongly repressed during the growth phase on glucose; once glucose concentration dropped below 5 nM, GFP expression was rapidly and strongly activated, exhibiting a dynamic range of 12.5-fold when the carbon source was switched to sucrose. Notably, dynamic repression of GFP expression was also achieved by coupling a full-length, antisense GFP cassette with the *SUC2* promoter in an RNAi capable strain (Williams et al., 2015b).

**FIGURE 3:**
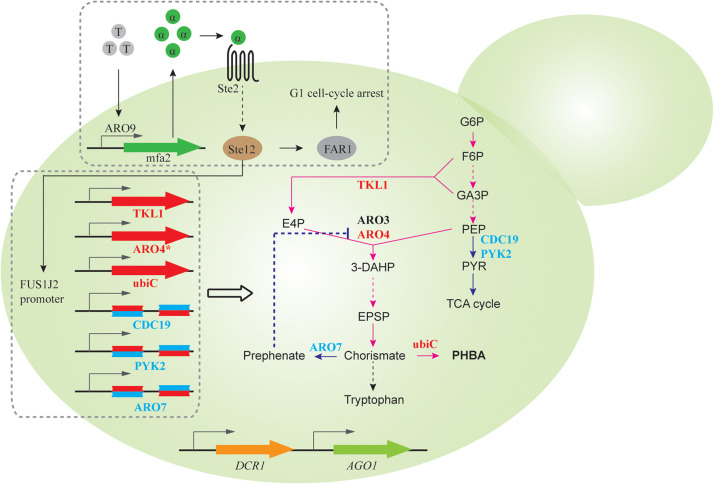
QS-linked, dynamic RNAi regulation during PHBA production. When the population density is high, the extracellular concentration of tryptophan-induced ARO9 promoter-dependent α-pheromone is high, which triggers the mitogen activated protein kinase (MAPK) phosphorylation cascade. The activated Ste12 transcription factor promotes the expression of FAR1, which leads to G1 cell-cycle arrest, and the expression of FUS1J2 promoter-mediated expression of *TKL1*, *ARO4*^K229L^ and *ubiC* gene, and RNAi cassettes targeting *CDC19*, *PYK2*, and *ARO7* gene, which enables the optimization of the PHBA pathway in the presence of a synthetic RNAi pathway.

Quorum sensing (QS) describes a widespread phenomenon that couples gene expression with population density (Miller and Bassler, 2001; Choudhary and Schmidt-Dannert, 2010), commonly achieved via cell-cell communications mediated by extracellular signaling molecules. QS can help to separate growth phase and production phase, when activation of target pathway genes and repression of competing metabolic activities are only initiated after sufficient biomass is accumulated. As a proof of concept, Williams et al. designed a synthetic QS circuit (Williams et al., 2013, 2015a), where an aromatic amino acid (i.e., tryptophan) induces expression and subsequent secretion of α-pheromone peptide. When extracellular pheromone accumulates to a certain threshold due to increasing cell densities, gene expression from the *FUS1J2* promoter is triggered and results in cell cycle arrest at the G1 phase (Figure 3). To achieve QS-regulated gene repression, RNAi constructs of *ARO7*, *PYK*, and *CDC19*, whose knockdown improves production of promoter para-hydroxybenzoic acid (PHBA) but impairs cell fitness, was expressed under the control of *FUS1J2* promoter (Figure 3). In an RNAi capable strain, QS-coupled RNAi regulation achieved 1.07 mM PHBA titer, which represents a 37-fold increase over the base strain without the QS circuit (Williams et al., 2015a). This work demonstrates the capability of QS-linked RNAi circuit in dynamic and multiplex gene repression for metabolic engineering.

### Genome-Scale Regulation

Genome-scale engineering creates microbial strain libraries for large-scale genotype-phenotype mapping (Si et al., 2015b). For *S. cerevisiae*, non-essential genes have been individually deleted for functional genomic studies (Winzeler et al., 1999; Giaever et al., 2002). But gene-knockout libraries are only available in certain laboratory strains, because it is traditionally tedious to replace each gene sequence with a selection marker via homologous recombination (Scherens and Goffeau, 2004). It is necessary to perform genome-wide screening in custom genetic background due to substantial phenotypic variations among different strains (Van Dijken et al., 2000). Thanks to its *trans-acting* nature, genome-scale knockdown is readily achieved by delivery of RNAi reagents to a select strain on episomal plasmids or via genomic integration. RNAi screening is therefore widely applied to improve a range of target traits in *S. cerevisiae*.

**FIGURE 4:**
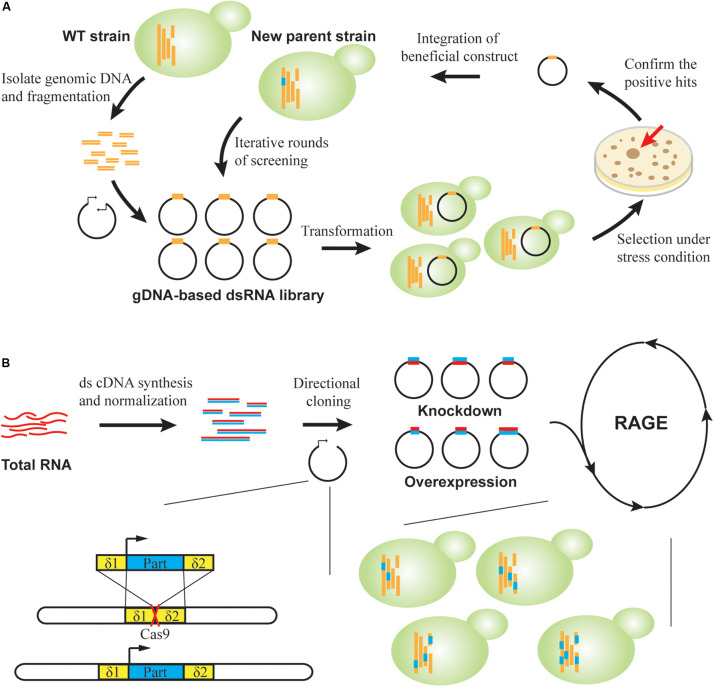
RNAi-assisted genome evolution in *S. cerevisiae*. **(A)** RAGE1.0. A dsRNA-encoding library is constructed by inserting genomic DNA fragments into convergent promoters. Strain libraries are created and screened on agar under selective pressure. For confirmed positive clones, corresponding beneficial RNA cassettes are genomically integrated to generate new parents for a subsequent round of selection. **(B)** RAGE2.0. Directional cloning creates overexpression and knockdown part libraries in a single step from cDNA libraries. Modulation parts are flanked by homologous δ sequences to direct multiplex genomic integration, which is facilitated by CRISPR-Cas. A standardized process of library creation and screening permits robotic iterations for directed genome evolution.

Using a long dsRNA library constructed with digested genomic DNA and convergent promoters, we performed the first genome-scale RNAi screening in *S. cerevisiae* (Si et al., 2014). We first validated the screening process by identification of known and new knockdown suppressors (*ret1*, *say1*, *ssa1*, *cst6*, and *mlp2*) of *yku70*Δ mutation, which causes yeast growth arrest at elevated temperature. Notably, *RET1* is an essential gene and hence cannot be identified using gene deletion collections. Moreover, inspired by directed protein evolution, we performed iterative rounds of RNAi screening for continuous improvement of acetic acid tolerance via accumulation of beneficial silencing cassettes, one at a time in each round (Figure 4A). Using this RNAi-assisted genome evolution (RAGE) method, we identified three knockdown targets (*ptc6*, *ypr084w*, and tRNA^Val(AAC)^) that synergistically enable yeast to grow in the presence of 1.0% (v/v) HAc. Similarly, the genomic DNA-derived RAGE library was applied to identify *siz1* as a new gene knockdown target to improve furfural tolerance in *S. cerevisiae* (Xiao and Zhao, 2014).

To achieve multi-mode, genome-scale engineering, we designed RAGE 2.0 libraries that permitted genome-wide overexpression and knockdown screening in a single step (Si et al., 2017; Figure 4B). Briefly, a normalized, full-length-enriched cDNA library was first constructed to cover >92% of yeast genes. To the 5′ and 3′ ends of cDNA molecules, we attached two different adaptor sequences that allowed directional cloning of cDNAs under a strong constitutive promoter P_TEF1_. Therefore, gene overexpression was achieved when cDNA molecules were transcribed in the sense direction, whereas gene repression was achieved through transcription of antisense RNAs in the presence of a heterologous RNAi pathway. Using this collection of modulation parts, both gene overexpression and knockdown targets were successfully identified in laboratory and industrial yeast strains for improved cellulase expression, isobutanol production, and xylose utilization (Si et al., 2017; HamediRad et al., 2018). Notably, in addition to isolating individual mutant strains, genome-wide mapping of gene–trait relations can be also achieved. We targeted glycerol substrate utilization, and improved mutants were enriched during serial transfer in glycerol media. Before and after growth enrichment, NGS was applied to track frequency changes of individual modulation parts, which was used to calculate enrichment ratios of corresponding mutants. In this way, the functional impact of every RAGE2.0 overexpression or knockdown mutation on glycerol utilization was quantified (Si et al., 2017).

Furthermore, the standardized and modular design of RAGE2.0 parts enabled accumulation of multiplex, genome-wide mutations in an evolving yeast genome (Si et al., 2017). We utilized the yeast genomic δ sites as landing pads for integrating multiple parts, where genomic integration efficiency was greatly enhanced by introduction of specific double-stranded breaks within δ sequences using the clustered regularly interspaced short palindromic repeats (CRISPR)/CRISPR-associated (Cas) system (Figure 4B). We then designed a standardized process where strain library creation and screening can be iteratively and robotically performed. After three rounds of selection under increasing HAc concentrations of 0.9, 1.0, and 1.1% (v/v), a mutant strain harboring at least 26 different modulation parts was isolated, indicating the success in creating multiplex genomic diversity. This strain was able to ferment glucose into ethanol in 1.1% (v/v) HAc media, which is the highest reported HAc tolerance to the best of our knowledge.

Alternatively, the Alper group pioneered in creating cDNA-derived RNAi libraries for tunable, genome-wide screening in *S. cerevisiae* (Crook et al., 2016). Using this library, Hsp70 family proteins were identified as key regulators of isobutanol tolerance. Reduced expression of these proteins increased biomass accumulation by 64% under 12 g/L isobutanol stress. Moreover, two rounds of screening identified two RNAi reagents targeting alcohol dehydrogenase and enolase. When combined, these two mutations exhibited a 31-fold improvement in cell growth in the presence of 10 g/L 1-butanol. Furthermore, reduced expression of the ribosome-associated chaperone Ssb1p was discovered to confer a 52% increase in lactic acid tolerance using the same RNAi library (Lee et al., 2016).

In a recent study, the Nielsen group integrated RNAi screening, microfluidic sorting, and Cas9-mediated genome recombineering to establish a workflow for identification and optimization of multiple gene targets (Wang et al., 2019). Two RNAi libraries were constructed, whereas the genomic DNA-derived library and full-length cDNA-derived library exhibited low and high knockdown efficiencies, respectively. Using droplet microfluidics, 1,500,000 cells covering ∼243,000 knockdown effectors were sorted and enriched in three rounds for improved α-amylase secretion, and genes involved in cellular metabolism, protein modification and degradation, and cell cycle were found to exert substantial impact on recombinant protein production.

Taken together, a variety of genome-scale RNAi libraries were created to screen a wide range of industrially relevant phenotypes in *S. cerevisiae*. These studies demonstrated several unique advantages. First, unlike gene deletion collections that are available only in certain laboratory strains (Scherens and Goffeau, 2004), genome-wide RNAi libraries can be readily introduced in a custom strain background, such as CEN.PK (Si et al., 2014, 2017; Wang et al., 2019), BY4741 (Xiao and Zhao, 2014; Crook et al., 2016; Lee et al., 2016), and industrial strains (Si et al., 2017; HamediRad et al., 2018). Notably, this feature enables the use of directed evolution strategies in genome-wide engineering, whereby beneficial mutations can be continuously accumulated in an evolving yeast genome (Si et al., 2014). Second, the multiplex nature of RNAi silencing permits simultaneous modulation of many gene targets, whose synergistic actions may lead to superior traits (Si et al., 2014, 2017; Wang et al., 2019). Third, mediating mRNA degradation, RNAi is particularly suited to screen gene silencing targets in polyploid industrial strains, whereby multiple gene copies often produce the same transcript (Si et al., 2017; HamediRad et al., 2018). Fourth, the use of RNAi expands the range of gene regulation in genome-scale screening, whereby not only various repression levels can be achieved by using different RNAi designs (Wang et al., 2019), but also inclusion of both overexpression and knockdown mutations in a single genome-wide library has been realized (Si et al., 2017).

On the other hand, there are certain limitations of genome-scale RNAi screening in *S. cerevisiae*. Due to the random nature of genomic DNA and cDNA-derived libraries, it is essential to perform necessary quality control to ensure sufficient genomic coverage and representation are achieved (Si et al., 2017). Also, due to the prevalence of off-target effects (Jackson et al., 2003), RNAi targets identified from large-scale screening need to be further confirmed by complementary approaches. For example, one can investigate whether the observed phenotype can be generated by a second RNAi reagent designed to target a distinct region of the same gene relative to the isolated RNAi (Si et al., 2014), as it is highly unlikely that two independent RNAi sequences exhibit the same off-target effect. Moreover, most RNAi screening studies in *S. cerevisiae* target “easy-to-screen” phenotypes such as chemical tolerance (Si et al., 2014, 2017; Xiao and Zhao, 2014; Crook et al., 2016; Lee et al., 2016) and substrate utilization (Si et al., 2017; HamediRad et al., 2018), because desirable mutant strains can be facilely identified by growth-based selection or enrichment. The integration of robotics and microfluidics will help to expand the scope of phenotypes that can be engineered using genome-scale RNAi screening (Si et al., 2017; Wang et al., 2019). Finally, for multiplex gene silencing (Si et al., 2017), simultaneous introduction of many RNAi reagents may saturate the RNAi machinery and resulted in inefficient silencing of individual targets, but such complication has not been rigorously assessed. Also, it is inherent challenging to speculate how multiple mutations may confer an improved trait, so that mechanistic studies are necessary to analyze the functional impact of single mutations and their interactions.

## Conclusion and Future Directions

RNA interference has long been a versatile tool for studying eukaryotic functional genomics studies but is absent from *S. cerevisiae.* Recent advances have greatly expanded the toolkit and application of RNAi in this model yeast, exhibiting many advantages including efficiency, specificity, graded and dynamic repression, genome-scale coverage, and multiplex targeting. Currently, a few groups have been routinely utilizing RNAi as a functional genomics and strain engineering tool in *S. cerevisiae*, although other potential applications such as genetic circuit design (Purcell et al., 2018) and gene delivery (Duman-Scheel, 2019) are also explored. One major factor limiting a wider use of RNAi by the *S. cerevisiae* communities is the availability of ample gene regulation tools in this model organism. Other gene repression methods include but are not limited to promoter engineering (Nevoigt et al., 2006), mRNA destabilization via 3′ insertion (Breslow et al., 2008), and CRISPR interference (CRISPRi) (Gilbert et al., 2013). However, the ease of creating genome-wide libraries directly from genomic DNA and cDNA molecules makes RNAi an attractive tool even in the era of CRISPR/Cas. Creating genome-wide deletion libraries by CRISPR/Cas requires genome sequencing data, algorithm to predict and rank guide RNA (gRNA) sequences, as well as introduction of homologous repair donors for efficient genome editing (Bao et al., 2018; Sharon et al., 2018). Using protein fusions between nuclease-dead mutants of Cas9 (dCas9) and transcriptional repressors, CRISPRi was successfully developed for genome-scale reduction-of-function screening in *S. cerevisiae* (Lian et al., 2019). Many prerequisites of CRISPR/Cas are not always available in polyploid industrial strains, but they do not limit the use of RNAi (HamediRad et al., 2018). Nonetheless, the ability to prototype gRNA collections based on array-synthesized oligo pools confers a greater potential of CRISPRi over RNAi, because the coverage and efficacy of gRNA libraries may be improved in “design-build-test” iterations. Notably, because both genome-scale CRISRPi and RNAi screening are currently performed in pooled formats, so that the silencing efficiency and off-target effect of individual reagents (gRNA or siRNA) remains unknown, which warrants detailed characterization in future studies.

Certain cautions should be taken during the design and implementation of RNAi in *S. cerevisiae*. The length and targeting position of hairpin RNA or dsRNA cassettes exhibit substantial impact on RNAi efficiency (Crook et al., 2014, 2016; Si et al., 2014), but there is currently no design rules. In addition, the expression level and mRNA structure of target gene may affect RNAi efficacy. For example, inefficient repression of *ZWF1* was attributed to the interference from mRNA secondary structure (Williams et al., 2015a). Moreover, RNAi often suffers from off-target effects, which may be particularly severe due to longer dsRNA precursors in budding yeasts for siRNA production (Weinberg et al., 2011; Wilson and Doudna, 2013; Ipsaro and Joshua-Tor, 2015). Systematic experimental evaluation and computer-aided design may help to understand and avoid the abovementioned issues (Lagana et al., 2014).

To further improve RNAi tools in *S. cerevisiae*, a more potent RNAi pathway can be engineered. For example, Xrn1 orthologs were reported to be involved in multiple steps of RNAi in *Arabidopsis thaliana*, *Caenorhabditis elegans*, *Drosophila melanogaster*, and *Homo sapiens* (Souret et al., 2004; Orban and Izaurralde, 2005; Chatterjee et al., 2011; Lima et al., 2016). In a genetic selection to discover novel components of RNAi in *S. castellii*, Xrn1p was found to play multiple roles to enhance silencing efficiency, including affecting the ratio of different types of siRNA precursors and duplexes, increasing siRNA loading into Ago1p, and enhancing degradation of the passenger strand (Getz et al., 2019). It is interesting to see if introduction of *S. castellii* Xrn1p can improve RNAi processing capabilities in *S. cerevisiae*, which may be limiting when multiplex RNAi regulation is performed (Si et al., 2017).

In addition to RNAi, other regulatory RNAs have been or can be potentially implemented for yeast strain engineering, such as CRISPR RNA, antisense non-coding RNAs, and tRNAs (David et al., 2006; Samanta et al., 2006; Donaldson and Saville, 2012; Si et al., 2015a; Bakowska-Zywicka et al., 2016). For example, antisense non-coding RNAs have been revealed for gene-specific transcriptional regulation, targeting *SER3* (Martens et al., 2004), *IME4* (Hongay et al., 2006), *PHO5* (Uhler et al., 2007), *PHO84* (Camblong et al., 2007, 2009; Castelnuovo et al., 2013), *GAL* (Houseley et al., 2008), *IME1* (Van Werven et al., 2012; Moretto et al., 2018), and *CDC28* (Nadal-Ribelles et al., 2014). Distinct mechanisms are employed, including mRNA interference, competition with transcriptional machinery, histone modification, and gene loop (Yamashita et al., 2016; Niederer et al., 2017; Till et al., 2018; Novačić et al., 2020). Systematic examination of antisense transcriptome will provide comprehensive insights into the mechanisms of antisense RNA-dependent gene regulation to guide engineering tool development (Huber et al., 2016; Nevers et al., 2018). With a deeper understanding, we envision the development of versatile RNA tools that are complementary to RNAi for facilitating strain engineering in *S. cerevisiae*.

## Author Contributions

YC and TS conceived the scope of this work. YC and EG drafted the manuscript. JZ and TS involved in revising and editing the manuscript. All authors approved the submitted version and agreed both to be personally accountable for the author’s own contributions and to ensure that questions related to the accuracy or integrity of any part of the work.

## Conflict of Interest

The authors declare that the research was conducted in the absence of any commercial or financial relationships that could be construed as a potential conflict of interest.
